# Molecular Detection of Tick-Borne Pathogens in Stray Dogs and *Rhipicephalus* *sanguineus* sensu lato Ticks from Bangkok, Thailand

**DOI:** 10.3390/pathogens10050561

**Published:** 2021-05-06

**Authors:** Thom Do, Pornkamol Phoosangwalthong, Ketsarin Kamyingkird, Chanya Kengradomkij, Wissanuwat Chimnoi, Tawin Inpankaew

**Affiliations:** 1 Department of Parasitology, Faculty of Veterinary Medicine, Kasetsart University, Bangkok 10900, Thailand; dothanhthom.t@ku.th (T.D.); pornkamol.ph@ku.th (P.P.); fvetksr@ku.ac.th (K.K.); fvetcyk@ku.ac.th (C.K.); fvetwic@ku.ac.th (W.C.); 2 Center for Agricultural Biotechnology, Kamphaeng Saen Campus, Kasetsart University, Nakhon Pathom 73140, Thailand; 3 Center of Excellence on Agricultural Biotechnology: (AG-BIO/PERDO-CHE), Bangkok 10900, Thailand

**Keywords:** canine tick-borne pathogens, stray dogs, temples, Thailand

## Abstract

Canine tick-borne pathogens (CTBPs) such as *Babesia vogeli*, *Ehrlichia canis*, *Anaplasma platys*, *Hepatozoon canis,* and *Mycoplasma haemocanis* are important pathogens in dogs worldwide. *Rhipicephalus sanguineus* sensu lato, the main vector of several CTBPs, is the most common tick species found on dogs in Thailand. The present study identified CTBPs in dogs and ticks infested dogs. Samples (360 dog blood samples and 85 individual ticks) were collected from stray dogs residing in 37 temples from 24 districts in Bangkok and screened for CTBPs using molecular techniques. The most common CTBP found infecting dogs in this study was *Ehrlichia canis* (38.3%) followed by *Mycoplasma haemocanis* (34.2%), *Hepatozoon canis* (19.7%), *Babesia vogeli* (18.1%), and *Anaplasma platys* (13.9%), respectively. Furthermore, *A. platys* (22.4%) was the most common CTBP in ticks followed by *M. haemocanis* (18.8%), *B. vogeli* (9.4%), *H. canis* (5.9%), and *E. canis* (2.4%), respectively. The detection of CTBPs from the present study highlights the potential risk of infections that may occur in stray dogs and their ticks residing in Bangkok temples. These findings underline the importance of performing active surveys to understand the complexity of distributions of CTBPs in dogs and their ticks in Thailand.

## 1. Introduction

The blood-feeding behavior of a wide range of arthropods, such as ticks and fleas, makes them important vectors of an array of viral, bacteria, and protozoan pathogens to humans and animals [1]. The brown dog tick, *R. sanguineus* s.l. is the most widely distributed tick species worldwide [2]. It is able to transmit several tick-borne pathogens to the host during a blood meal [3]. Some of these pathogens are transmitted to the subsequent tick developmental stages known as transstadial maintenance [4].

Canine tick-borne pathogens (CTBPs) including *Ehrlichia* spp., *Anaplasma* spp., hemotropic mycoplasma, *Babesia* spp. and *Hepatozoon* spp., widely affect canine health [5,6,7,8]. *Anaplasma* and *Ehrlichia* species are obligate intracellular Anaplasmataceae bacteria in animals [9] and are mostly detected in canids in tropical and subtropical areas [3]. There are at least three *Ehrlichia* species infecting dogs, namely *E. canis, E. chaffeensis* and *E. ewingii* [10], of which *E. canis* is the etiologically important agent of canine monocytic ehrlichiosis [11]. Furthermore, *A. phagocytophilum* and *A. platys* have been documented as the main causative agents of canine anaplasmosis in temperate zones and canine cyclic thrombocytopenia in tropical areas, respectively [10,11]. Other bacteria have been reported recently within the genus *Mycoplasma* including *Mycoplasma haemocanis* [12] and *Candidatus* Mycoplasma haematoparvum [13] causing a severe hemolytic syndrome in dogs. In addition, *Babesia* and *Hepatozoon* species are of the most widespread apicomplexan protozoan parasites causing severe diseases and sometimes deaths in infected dogs [14]. Specifically, at least four species of *Babesia* (*B. gibsoni*, *B. canis*, *B. rossi*, and *B. vogeli*) and two *Hepatozoon* species (*H. canis* and *H. americanum*) are agents of canine babesiosis and hepatozoonosis, respectively [9]. Most of the earlier mentioned CTBPs can be transmitted to other dogs by ticks, blood transfusion or dog fighting, except for *Hepatozoon* which is transmitted primarily through the ingestion of ticks containing mature *H. canis* oocysts [15].

In Southeast Asia, including Thailand, the presence of stray or neglected companion animals and the high popularity of dog ownership contribute to favorable conditions for tick development, leading to enhanced transmission of tick-borne pathogens [16]. In many Thai communities, owned dogs are allowed to roam freely outdoors. These animals can have a high risk of tick infestation and tick-borne infection when they get in contact with infected animals. The present study aimed to investigate the occurrence of the commonly reported CTBPs in stray dogs and in *R. sanguineus* s.l. ticks in the Bangkok metropolitan area.

## 2. Results

### 2.1. CTBPs Infection in Blood Samples

Of the 360 dogs, 275 (76.4%) were infected by at least one of the five pathogens. *E. canis*, *M. haemocanis*, *H. canis*, *B. vogeli*, and *A. platys* were detected in dogs with prevalence levels of 38.3% (138/360), 34.2% (123/360), 19.7% (71/360), 18.1% (65/360), and 13.9% (50/360), respectively. Co-infections were detected in 130 dogs (36.1%). Co-infection of CTBP was detected in 95 individuals (26.4%) for two CTBPs, of which *E. canis*/*M. haemocanis* accounted for 29.5% (28/95), followed by *E. canis*/*H. canis* 13.7% (13/95) and *M. haemocanis*/*H. canis* 10.5% (10/95), respectively. Co-infection by three and four pathogens were detected in 28 (7.8%) and 7 dogs (1.9%), respectively (Table 1).

### 2.2. CTBPs Infection in Tick Samples

The overall infection prevalence of ticks was 38.8% (33/85). *A. platys* was the most prevalent bacterium in ticks with 22.4% (19/85), followed by *M. haemocanis* 18.8% (16/85), *B. vogeli* 9.4% (8/85), *H. canis* 5.9% (5/85), and *E. canis* 2.4% (2/85), respectively. Co-infection by at least two pathogens was found in 11 (12.9%) samples, of which eight ticks (9.4%) were co-infected by two pathogens and three ticks (3.5%) positive for three CTBP, which were *A. platys*/*B. canis*/*H. canis* (1/85, 1.1%) and *A. platys*/*M. haemocanis*/*H. canis* (2/85, 2.3%) (Table 1).

### 2.3. Risk Factors Associated with CTBPs Infection

Dogs with tick infestation (*p* = 0.005, OR = 2.81, 95% CI: 1.38–5.73) were more likely to be infected with CTBP compared to the dogs without ticks. A significant difference was found between dog age groups and CTBPs. Specifically, *B. vogeli* infection (27.5%, OR = 2.27, 95% CI: 1.19–4.33) and *H. canis* infection (27.5%, OR = 2.27, 95% CI: 1.19–4.33) were significantly higher in puppies than juveniles and adults. However, there was no significant difference among age groups for *A. platys* (χ^2^ = 4.98, df = 2, *p* = 0.08), *E. canis* (χ^2^ = 2.44, df = 2, *p* = 0.29) and *M. haemocanis* (χ^2^ = 1.30, df = 2, *p* = 0.5) infection, respectively. Infestation by *R. sanguineus* (s.l.) significantly increased the risk of *E. canis* (OR = 3.24, 95% CI: 1.96–5.36) and *B. vogeli* infection (*p* = 0.02, OR = 2.06, 95% CI: 1.15–3.68) (Table 2).

### 2.4. Sequence Analysis

For each genus of TBP detected from the blood and tick samples, positive amplicons were subjected to sequencing and BLAST analysis. All obtained sequences for each pathogen originated from blood and tick samples shared 100% identity together and shared 99.7% identity with reported *M. haemocanis* (GenBank: KY117659, and KP715860), *H. canis* (GenBank: KU527126, MK091086, and KC138532) and *B. vogeli* (GenBank: MN823219, MK881091, and MH100721) isolates, respectively. For *Anaplasma* and *Ehrlichia* species, all positive amplicons shared 100% sequence identities with published isolates of *A. platys* (GenBank: LC428207) and *E. canis* (GenBank: KU765198), respectively. Representative sequences from the present study were submitted to Genbank under accession numbers MW406796–406800 (*M. haemocanis*), MW255597–255601 (*H. canis*), MW255605–255609 (*B. vogeli*), MW390801–390805 (*A. platys*), and MW382939–382940 (*E. canis*).

**Figure 1:**
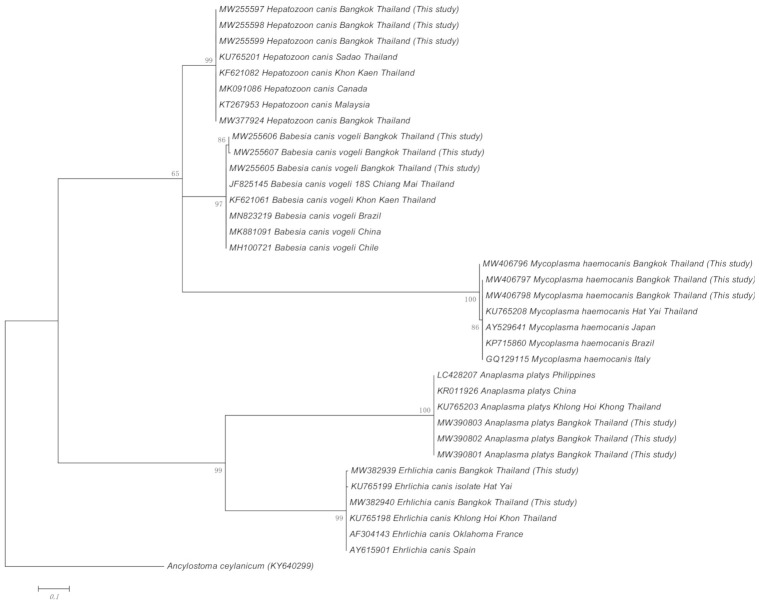
Phylogenetic tree of each CTBP sequences based on the 16S rRNA gene (*Mycoplasma*), the 18S rRNA gene (*Hepatozoon*, *Babesia*), the *groESL* gene (*Anaplasma)* and the *gltA* gene (*Ehrlichia*) obtained from this study using Maximum Likelihood method (Kimura-two-parameter model). Numbers at node represent percentage occurrences clades based on 1000 bootstrap replication of data; *Ancylostoma caninum* is provided as an outgroup species.

Representative sequences of each CTBP obtained from this study and previous reports were used to establish a phylogenetic tree. In the representing tree, *Ancylostoma ceylanicum* was used as outgroup species to the root tree. In the phylogenetic analysis for *H. canis*, *B. vogeli, M. haemocanis, A. platys,* and *E. canis* demonstrate that variability between the sequence of theses pathogens and those from other geographic regions are low. All the isolates of each species formed separate clades with a high bootstrap support (Figure 1). The *A. platys* and *E. canis* sequences were identical to the reference sequences, whereas a low degree of genetic variability (1–3 SNPs) was observed in *B. vogeli*, *H. canis*, and *M. haemocanis* sequences compared with their respective reference sequences (Supplementary Table S1). Therefore, it must be taken into account that low genetic polymorphism among species occurred in the current study.

## 3. Discussion

The documented rates of CTBPs in dogs varied in several epidemiological surveys conducted in different geographical areas in Thailand [11,17,18,19]; however, only a few studies of Vector-borne pathogens have been done based on vector. Our results indicated that CTBPs including *A. platys*, *B. vogeli*, *E. canis*, *M. haemocanis* and *H. canis* are endemic in the studied area.

The most PCR-detected tick-transmitted bacterium contracted by canines was *E. canis* (38.3%), the causative agent of canine monocytic ehrlichiosis. In agreement with this study, *E. canis* was the most prevalent tick-borne bacterium (21.5–36%) reported in northern Thailand [17,20,21]. In other Southeast Asian countries, the relatively high occurrence of *E. canis* is consistent with previous studies reporting 21.8%, 15.7%, 25.8%, 5.3%, 11.1%, and 36.2% in Cambodia [7], the Philippines [3], Vietnam, Singapore, Malaysia, and Indonesia [22], respectively. *E. canis* has been occasionally reported in humans causing human monocytic ehrlichiosis and considered as a minor zoonotic agent [23,24]. The occurrence of *E. canis* in ticks (2.4%) in this study suggested another potential risk of *E. canis* transmission from a tick to humans [25].

Canine hemotropic mycoplasma seems to have a worldwide distribution, though only restricted prevalence data are available based on molecular detection methods [26]. Three common species of *Mycoplasma* spp. in dogs (*Candidatus* Mycoplasma haematoparvum, *Candidatus* Mycoplasma haemominutum and *Mycoplasma haemocanis*) have been detected in Thailand [18]. They were considered as a new health threat to dogs observed with the highest frequency; however, they have been rarely confirmed by veterinary diagnosis since the first report in 2016. The *M. haemocanis* rate detected in stray dogs (34.2%) in present study should prompt greater awareness by veterinarians and physicians in isolated diagnosis and treatment since a specific antimicrobial therapy has been required in the treatment [27]. Hemotropic mycoplasmas have been deemed tick-borne pathogens since their transmission by ticks was initially proven in 1973 [28]. The finding of *M. haemocanis* in dogs and *R. sanguineus* s.l. suggests its vector competence for this pathogen. However, although *M. haemocanis* (18.8%) was detected in ticks in the present survey, it might have been simply part of the blood meal taken from the host. Thus, a further study on vectorial competence is needed to verify the relationship between hemotropic mycoplasma and ticks.

The occurrence of *B. vogeli* and *H. canis* has been reported in animals from Southeast Asia [3,7,22,29]. The difference observed in the present study with respect to others in Asia could be attributed to the difference in the number of dogs and the selection criteria, the sampling size, the geographical area, the sampling season, and the target gene [3,18]. A large-scale survey conducted in East and Southeast Asia reported that none of the owned dogs from Thailand and their ticks tested positive for *Babesia* spp. or *Mycoplasma* spp. [22,30]. Interestingly, both infections were common in the stray dog population (18.1% and 34.2%, respectively) and tick samples (9.4% and 18.8%, respectively) collected in the present study. By sharing a common environment with humans and other domesticated animals, this result should alert people to the risk of CTBP infection transmitted from these stray animals and their parasitic arthropods.

The brown dog tick, *R. sanguineus* s.l. is among the most important arthropod vector accountable for the transmission of several pathogens causing babesiosis and ehrlichiosis in Asia [3,22]. All the ticks collected from dogs in the present study were *R. sanguineus* s.l. which has been reported as the most common tick species infesting dogs in Thailand with the prevalence over 90% [31]. All the investigated TBPs were found in examined vectors. Surprisingly, of the CTBPs detected, *A. platys* was the most common pathogen found in ticks (22.4%) but was the least common species found in blood samples (13.9%). On the other hand, *E. canis* had the highest detection in blood samples (38.3%) but the lowest occurrence in ticks (2.4%) in the present study. These results were different from to that in a previous study which reported that *E. canis* was the most detected pathogen in blood and tick samples [3]. The difference between our results and those of other studies could have been due to the low sample size of ticks examined. A further survey with a greater sample would overcome this restriction and should provide a more definitive conclusion of the relationship between ticks and different CTBPs. Furthermore, the detection rate of the CTBPs seemed higher in the blood samples compared to the tick samples in the present study, which could be explained by during the appropriate period of tick development, one dog can be infested by several ticks that might have maintained several pathogens or dogs might have been infected from earlier tick infestation; therefore, the individual dog will certainly be more likely infected with different CTBPs. In addition, the higher occurrence rate of CTBPs in the hosts than those in the vectors might have been due to the presence of other potential routes of CTBP transmission in the investigated dogs such as by blood exchange in fighting dogs [32]. Other multiple factors such as the different biological cycle of each single CTBP in their host or the distinct behavior of the CTBP during the reactivate process in the grace period could also be factors to affect the number of pathogens in ticks and in the host [33].

Concurrent infection with two or more CTBPs was frequent in the studied dogs. It was tested in 130 (36.1%) individuals, of which, *E. canis* and *M. haemocanis* constituted the most common co-infection pattern. Given that the co-infection of multiple vector-borne pathogens in the same canine is common in tropical areas [34,35], this might have been due to the high diversity of both infectious agents and vectors in such regions with poor access to veterinary care [16]. The occurrence of co-infection may cause greater pathogenicity whereby greater variable signs were exhibited by the affected dogs, resulting in a more challenging diagnosis. Our finding was in agreement with previous statements on the importance of testing for more than one CTBP [3,7]. Co-infection with at least two CTBPs was also frequently observed in examined ticks. It was found in 11 (12.9%) ticks, with the combination of *A. platys* and *M. haemocanis* being the most common co-infection pattern (3.5%). These findings re-enforced the elucidation of multiple infections of CTBPs in dogs resulted from transmission with multiple pathogens by the same tick or individual pathogens by different ticks [36]. In fact, the current analysis of some host attributes showed that tick-infested dogs had a significantly higher tendency to become infected with CTBPs. The statistical analysis revealed that tick-infested dogs were 2.81 times (OR = 2.81, 95% CI 1.38–5.73) at higher risk of CTBP infection compared to non-infested dogs. Previously, some reports similarly concluded that the likelihood of a dog becoming in contact with a vector-borne pathogen in a given area was greatly influenced by the vector population density as well as by the prevalence of the infection within the vector population [37]. In addition, there was a higher tendency for puppies to become infected with *B. vogeli* and *H. canis* in the current study. This phenomenon has been previously attributed to their greater susceptibility to tick infestation and heavier tick burden than older dogs [38]. In addition, stray dogs with their roaming behavior can spread ticks from one place to another, thereby playing an important role in the spread of CTBPs [3].

This study conducted a molecular survey of *A. platys*, *B. vogeli*, *E. canis*, and *H. canis* in stray dogs in Bangkok, Thailand. Our findings revealed that *A. platys*, *B. vogeli*, *E. canis*, *M. haemocanis*, and *H. canis* are endemic in the studied area, of which *E. canis* was the most PCR-detected tick-transmitted pathogen. Concurrent infection with two or more CTBPs was frequent in the studied dogs in which *E. canis* and *M. haemocanis* constituted was the most common co-infection pattern. *R. sanguineus* s.l. is the most common tick species infesting in dogs in the studied areas and in Thailand. This finding is important in molecular phylogenetic studies by contributing to the literature about the canine tick-borne pathogen epidemiology in Thailand.

## 4. Materials and Methods

### 4.1. Data on Sample Collection

**Figure 2:**
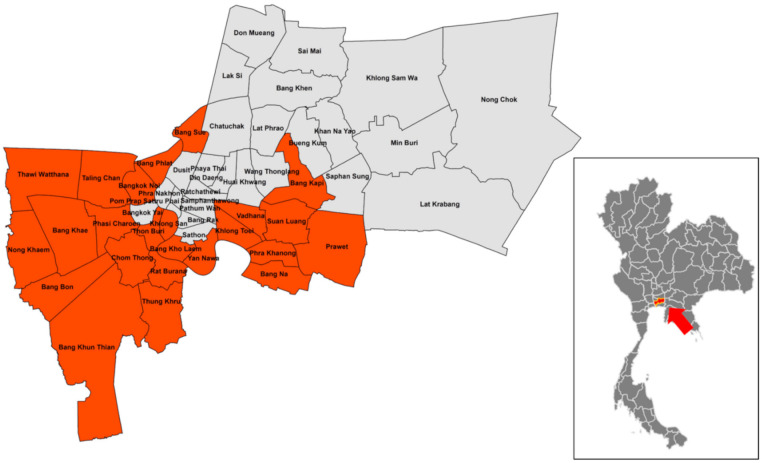
Map of study area in Thailand. The arrow in the smaller map indicates the location of Bangkok. The orange-highlighting in the larger map of Bangkok shows the sampling area.

This study included districts in Bangkok located in central Thailand (13°45′ N and 100°30′ E) characterized by an average annual temperature of 29.7 °C and a monthly rainfall of 205.4 mm (Figure 2). Ticks and blood samples were collected from stray dogs residing in 37 monasteries from 24 districts in the Bangkok metropolitan area from March to June 2015. Each dog was humanely manually restrained and at least 1 mL of blood was drawn from the jugular vein into vacutainer tubes containing ethylenediamine tetra-acetic acid anticoagulant using a 3 mL syringe with a 23G needle performed by a qualified veterinary technician. Blood samples were kept in a freezer (−20 °C) in the Department of Parasitology, Faculty of Veterinary Medicine, Kasetsart University, Bangkok, Thailand, until retrieval for further laboratory investigations. The whole body of each dog was carefully inspected and adult ticks attached to the dog were collected and placed in 1.5 mL tubes containing 70% ethanol for later morphological identification. Ticks were identified under a stereomicroscope to the level of species [39].

Data of the enrolled dogs were determined and gathered by a qualified veterinarian and classified into: age (puppy < 1 year, juvenile 1–3 years, adults > 3 years), sex, and tick infestation (yes/no). In total, 360 dog blood samples were included, consisting of 161 (44.7%) from males and 199 (55.3%) from females. All dogs were Thai local and mixed breed with an age distribution of 91 (25.3%) puppies, 110 (30.6%) juveniles, and 159 (44.2%) adults. The most common clinical manifestations of vector-borne infection were inappetence, fever, lethargy, pale mucus membranes, jaundice, and vomiting, but not all dogs showed clinical signs at the time of sampling. Only 85 (23.6%) of the dogs carried ticks at the time of blood collection. All ticks were morphologically identified as *R. sanguineus* s.l. All the procedures were carried out according to ethical guidelines for the use of animal samples permitted by the Animal Ethics Committee of Kasetsart University, Bangkok, Thailand (ACKU60-VET006).

### 4.2. PCR Detection and Sequence Analysis

Tick samples were prepared before DNA extraction. Briefly, after removal of the ethanol by washing with phosphate-buffer saline, the ticks were ground thoroughly using a sterilized micropestle and then the tubes were placed in a boiling water bath with Proteinase K for 12 h. Genomic DNA from blood samples and ticks were extracted using an E.Z.N.A.^®^ Blood DNA Mini Kit and an E.Z.N.A.^®^ Tissue DNA Kit (Omega Biotek Inc., Norcross, GA, USA) following the manufacturer’s instruction. The concentration of extracted DNA was measured at 260/230 nm using a BioSpectrometer (Eppendorf AG, Hamburg, Germany). Subsequently, conventional PCR was used to test the DNA samples for the presence of *A. platys*, *E. canis*, *Babesia* spp., *Hepatozoon* spp., and *Mycoplasma* spp. (Table 3). All DNA amplifications were performed in a 25 µL reaction mixture consisting of distilled deionized water, 1 µL of template DNA, 10 pmol of each primer, 10 mM of each deoxynucleotide triphosphate, 2.5 µL of 10× buffer, and 0.13 unit of Taq DNA polymerase (BioFact^TM^, Daejeon, South Korea). Amplifications were performed using an Eppendorf MasterCycler Nexus Gradient Thermal Cycler (Eppendorf AG, Hamburg, Germany) under the previously described conditions with some modification (Table 3). A negative control (distilled deionized water) and positive controls (positive DNA of each pathogen extracted from blood of infected dogs) were used in each PCR reaction. The PCR products were checked using electrophoresis in 1.5% agarose gel (LE agarose, Thermo Fisher Scientific, Waltham, MA, USA) and TAE (Tris-acetate-EDTA) buffer.

For sequence analysis, selected positive amplicons were snipped from the gel and purified using a FavorPrep^TM^ GEL/PCR Purification Kit (Favorgen, Prima Scientific Co., Bangkok, Thailand). Subsequently, the purified product was submitted for Sanger DNA sequencing (Macrogen, Seoul, Korea). The raw nucleotide sequences and chromatograms were viewed using the BioEdit version 7.2 (www.mbio.ncsu.edu/BioEdit/bioedit.html, accessed on 26 April 2021) and FinchTV 1.4.0 (Geospiza, Inc., Seattle, WA, USA) programs and the sequences were aligned and analyzed using the Clustal W software version 2.0 [40]. The sequences were compared with published isolates using the Basic Local Alignment Search Tool (BLAST) of the U.S. National Center for Biotechnology Information (https://blast.ncbi.nlm.nih.gov/Blast.cgi, accessed on 26 April 2021) to determine the *Anaplasma*, *Babesia*, *Ehrlichia*, *Hepatozoon,* and *Mycoplasma* species.

### 4.3. Phylogenetic Analysis

The genetic relationship of each CTBP isolate from this study and those from other regions of Thailand and the world was established by phylogenetic analyses using MEGA X software (https://www.megasoftware.net, accessed on 26 April 2021). The maximum-likelihood method with Kimura-two-parameter model was employed to construct the phylogenetic trees. Bootstrap analysis with 1000 replication was set to estimate the confidence of the branching patterns of the tree.

### 4.4. Statistical Analysis

The statistical association between the detection rate of CVBPs obtained by PCR and the categorical variables regarding age, sex and tick infestation were analyzed using a chi-square test (cell frequencies > 5) or Fisher’s exact test (cell frequencies ≤ 5). Any parameters statistically linked to positive PCR results were used in a logistic regression model with an odds ratio (OR) to evaluate the independent risk factors associated with infection. The statistically significant level was established at *p* ≤ 0.05. Data were analyzed using the R software [41].

## 5. Conclusions

The current data have shown the potential risk of CTBPs in stray dogs residing in the temples studied in Bangkok. The infection of CTBPs in the host population along with the detection of these pathogens in *R. sanguineus* s.l. in sampling areas supported the vector role in the transmission of these CTBPs in this region. These results emphasize the need for testing multiple CTBPs in dogs suspected of infection to facilitate appropriate treatment and to prevent the risk of transmission of CTBPs to animal.

## Figures and Tables

**Table 1:** The occurrence rate of tick-borne pathogens in the studied blood and tick samples.

Pathogen	Dogs Infected n = 360 (%)	Ticks Infected n = 85 (%)
CTBP total	275 (76.4)	33 (38.8)
*A. platys*	50 (13.9)	19 (22.4)
*E. canis*	138 (38.3)	2 (2.4)
*B. vogeli*	65 (18.1)	8 (9.4)
*M. haemocanis*	123 (34.2)	16 (18.8)
*H. canis*	71 (19.7)	5 (5.9)
1 CTBP species	145 (40.3)	22 (25.9)
*A. platys*	13 (3.6)	7 (8.2)
*E. canis*	50 (13.9)	0
*B. vogeli*	20 (5.6)	5 (5.9)
*M. haemocanis*	46 (12.8)	9 (10.6)
*H. canis*	16 (4.4)	1 (1.2)
2 CTBP species	95 (26.4)	8 (9.4)
*A. platys + E. canis*	10 (2.78)	2 (2.4)
*A. platys + B*. *vogeli*	3 (0.9)	0
*A. platys + M. haemocanis*	10 (2.8)	3 (3.5)
*A. platys + H. canis*	1 (0.3)	1 (1.2)
*E. canis + B. vogeli*	9 (2.5)	0
*E. canis + M. haemocanis*	28 (7.8)	0
*E. canis + H. canis*	13 (3.6)	0
*B. vogeli + M. haemocanis*	7 (1.9)	2 (2.4)
*B. vogeli + H. canis*	4 (1.1)	0
*M. haemocanis*. *+ H. canis*	10 (2.9)	0
3 CTBP species	28 (7.8)	3 (3.5)
*A. platys + E. canis + B. vogeli*	1 (0.3)	0
*A. platys + E. canis + M. haemocanis*	1 (0.3)	0
*A. platys + E. canis + H. canis*	2 (0.6)	0
*A. platys + B. vogeli + M. haemocanis*	1 (0.3)	0
*A. platys + B. vogeli + H. canis*	2 (0.6)	1 (1.2)
*A. platys + M. haemocanis + H. canis*	3 (0.8)	2 (2.4)
*E. canis + B. vogeli + M. haemocanis*	5 (1.4)	0
*E. canis + B. vogeli + H. canis*	7 (1.9)	0
*E. canis + M. haemocanis + H. canis*	5 (1.4)	0
*B. vogeli + M. haemocanis + H. canis*	1 (0.3)	0
4 CTBP species	7 (1.9)	0
*A. platys + E. canis + B. vogeli + M. haemocanis*	0	0
*A. platys + E. canis + B. vogeli + H. canis*	1 (0.3)	0
*A. platys + E. canis + M. haemocanis + H. canis*	1 (0.3)	0
*A. platys + B. vogeli + M. haemocanis + H. canis*	1 (0.3)	0
*E. canis + B. vogeli + M. haemocanis + H. canis*	4 (1.1)	0
Mixed CTBP	130 (6.1)	11 (12.9)

**Table 2:** Risk factors associated with the canine tick-borne pathogen detected on the blood test.

Attribute	Total Number n = 360	Number of Positive Dogs					
		Any of the Pathogens	*Anaplasma platys*	*Ehrlichia canis*	*Babesia vogeli*	*Mycoplasma haemocanis*	*Hepatozoon canis*
**Age category (year)**							
<1	91 (25.3)	74 (81.3)	19 (20.9)	41 (45.1)	25 (27.5) **	27 (29.7)	25 (27.5) **
1–3	110 (30.6)	85 (77.3)	13 (11.8)	41 (37.3)	18 (16.4)	41 (37.3)	24 (21.8)
>3	159 (44.2)	116 (72.9)	18 (11.3)	56 (35.2)	22 (13.8)	55 (34.6)	22 (13.8)
**Sex**							
Male	161 (44.7)	122 (75.8)	23 (14.3)	57 (35.4)	26 (16.1)	55 (34.2)	32 (19.9)
Female	199 (55.3)	153 (76.9)	27 (13.6)	81 (40.7)	39 (19.6)	68 (34.2)	39 (19.6)
**Ticks**							
Presence	85 (23.6)	75 (88.2) **	11 (12.9)	51 (60) **	23 (27.1) **	31 (36.5)	22 (25.9)
Absence	275 (76.4)	200 (72.7)	39 (14.2)	87 (31.6)	42 (15.3)	92 (33.5)	49 (17.8)
Total	360	275 (76.4)	50 (13.9)	138 (38.3)	65 (18.1)	123 (34.2)	71 (19.7)

** Statistically significant difference (*p* < 0.05).

**Table 3:** Sequences of primer sets used for canine tick- borne pathogens detection.

Pathogen	Oligonucleotide Sequences (5′–3′)	Product Size (bp)	PCR Protocol	Reference
*Anaplasma platys (groESL)*	F: AAGGCGAAAGAAGCAGTCTTA R: CATAGTCTGAAGTGGAGGAC	724	95 °C for 5 min initial denaturation, followed by 35 cycles of 95 °C for 15 s, 50 °C for 30 s, 72 °C for 30 s, then 72 °C for 2 min for the final elongation	[42]
*Ehrlichia canis (gltA)*	F: TTATCTGTTTATGTTATATAAGC R: CAGTACCTATGCATATCAATCC	1251	94 °C for 2 min initial denaturation, followed by 44 cycles of 94 °C for 30 s, 53 °C for 60 s, 68 °C for 60 s, then 68 °C for 3 min for the final elongation	[43]
*Babesia* spp. (18S rRNA)	F: GTTTCTGMCCCATCAGCTTGAC R: CAAGACAAAAGTCTGCTTGAAAC	422–440	94 °C for 3 min initial denaturation, followed by 35 cycles of 94 °C for 30 s, 50 °C for 30 s, 72 °C for 1 min, then 72 °C for 5 min for the final elongation	[44]
*Hepatozoon* spp. (18S rRNA)	F: ATACATGAGCAAAATCTCAAC R: CTTATTATTCCATGCTGCAG	666	94 °C for 3 min initial denaturation, followed by 34 cycles of 95 °C for 30 s, 50 °C for 30 s, 72 °C for 1 min, then 72 °C for 5 min for the final elongation	[45]
*Mycoplasma* spp. (16S rRNA)	F: ATACGGCCCATATTCCTACG R: TGCTCCACCACTTGTTCA	595	94 °C for 5 min initial denaturation, followed by 40 cycles of 95 °C for 30 s, 60 °C for 30 s, 72 °C for 30 s, then 72 °C for 10 min for the final elongation	[46]

Abbreviations: F: Forward, R: Reverse, *groESL*: The heat shock protein gene, *gltA*: The citrate synthase gene.

## Data Availability

Data is contained within the article and Supplementary Materials.

## Supplementary Materials

The following are available online at https://www.mdpi.com/article/10.3390/pathogens10050561/s1, Table S1: Multiple sequence alignment analysis showing single nucleotide gene polymorphisms (SNPs) found within, *A. platys*, *B. vogeli*, *E. canis*, *H. canis*, and *M. haemocanis* compared with reference sequences.
