# Supplementary material for: Molecular Detection of Tick-Borne Pathogens in Stray Dogs and Rhipicephalus sanguineus sensu lato Ticks from Bangkok, Thailand
Source: Pathogens. 2021 May 6;10(5):561. doi: 10.3390/pathogens10050561 (PMC8148546; doi:10.3390/pathogens10050561)
Supplement: Supplementary file 1 [file pathogens-10-00561-s001.zip › Supplementary Table S1.pdf]

Supplemental Table S1. Multiple sequence alignment analysis showing single nucleotide gene polymorphisms (SNPs) found within, *A. platys*, *B. vogeli*, *E. canis*, *H. canis* and *M. haemocanis* compared with reference sequences.

Assemblage/ Sub-assemblage	GenBank Accession no.	No. of isolates	Nucleotide at position*					
			169	345	458	633	750	943
A. platys								
Reference	LC428207		*	*	*	*	NA	NA
Dogs	MW390801	5	*	*	*	*	NA	NA
B. vogeli								
Reference	MN823219		A	*	*	*	NA	NA
Dogs	MW255605	5	G	*	*	*	NA	NA
E. canis								
Reference	KU765198		*	*	*	*	*	*
Dogs	MW382939	2	*	*	*	*	*	*
H. canis								
Reference	KU527126		*	T	*	T	NA	NA
Dogs	MW255598	5	*	C	*	C	NA	NA
M. haemocanis								
Reference	KY117659		*	*	A	*	C	A
Dogs	MW406796	5	*	*	G	*	T	G

Key: Asterisks (*) indicate nucleotide identity with the reference sequence. NA not available

Nucleotide positions are numbered according to the reference *A. platys groESL* partial sequence (GenBank accession number LC428207), with the first nucleotide as position 27, *B. vogeli* 18S rRNA partial sequence (GenBank accession number LC437493) with the first nucleotide as position 166, *E. canis gltA* partial sequence (GenBank accession number DQ220289), with the first nucleotide as position 1, *H. canis* 18S rRNA partial sequence (GenBank accession number DQ220289), with the first nucleotide as position 60 and *M. haemocanis* 16S rRNA partial sequence (GenBank accession number KP890051), with the first nucleotide as position 124.
